# Case Report: Ultrasound-guided thrombin injection for the treatment of radial artery pseudoaneurysm after percutaneous coronary intervention

**DOI:** 10.3389/fcvm.2026.1753285

**Published:** 2026-02-09

**Authors:** Xiaoyan Li, Guoqing Qi, Liye Wei

**Affiliations:** 1 Department of Cardiology, The First Hospital of Hebei Medical University, Shijiazhuang, Hebei, China; 2 Department of Cardiology, Beijing Genertec Aerospace Hospital, Beijing, China

**Keywords:** complication, percutaneous coronary intervention, radial artery pseudoaneurysm, thrombosis, ultrasound-guided thrombin injection

## Abstract

**Background:**

Radial artery pseudoaneurysm (RAP) is an uncommon but significant complication after transradial coronary procedures. This case demonstrates the efficacy of ultrasound-guided thrombin injection (UGTI) as a minimally invasive treatment.

**Case presentation:**

A 65-year-old Chinese male developed a RAP two days after percutaneous coronary intervention. Diagnosis was confirmed by Doppler ultrasound. UGTI was performed, injecting 1.5 mL of thrombin. Complete thrombosis occurred within 30 s, with sustained occlusion confirmed at 24-hour follow-up.

**Conclusions:**

UGTI is a highly effective and safe first-line treatment for iatrogenic RAP, enabling rapid resolution and avoiding the need for surgery. It represents a superior minimally invasive strategy for managing this vascular complication.

## Introduction

Radial artery access has gained widespread preference over femoral access for coronary angiography and percutaneous coronary intervention (PCI) due to its association with fewer vascular complications, reduced bleeding risks, and enhanced patient comfort (1). Nonetheless, vascular complications such as radial artery pseudoaneurysm (RAP) may still occur, particularly among elderly patients with comorbidities such as atherosclerosis and hypertension (2, 3). RAP typically manifests as a painful, pulsatile mass at the puncture site and may lead to serious sequelae including nerve compression, distal ischemia, or rupture if left untreated (4, 5).

The management of RAP has evolved from primarily surgical repair, once considered a definitive treatment, to a spectrum of strategies including prolonged external compression, ultrasound-guided thrombin injection (UGTI), and endovascular exclusion techniques (6, 7). Small-neck pseudoaneurysms are often amenable to conservative or minimally invasive approaches, whereas those with wider necks or symptoms of compression may require more aggressive intervention (8, 9). UGTI, initially established for femoral pseudoaneurysms, has been increasingly applied in radial artery cases due to its minimally invasive nature and high technical success rates (10). However, its safety profile necessitates careful patient selection and operator expertise to avoid complications such as distal thromboembolism or radial artery occlusion (11). This case report illustrates the successful application of UGTI for a small-neck RAP in a 65-year-old male following coronary PCI, and discusses its role within contemporary treatment algorithms.

## Case presentation

**Figure 1:**
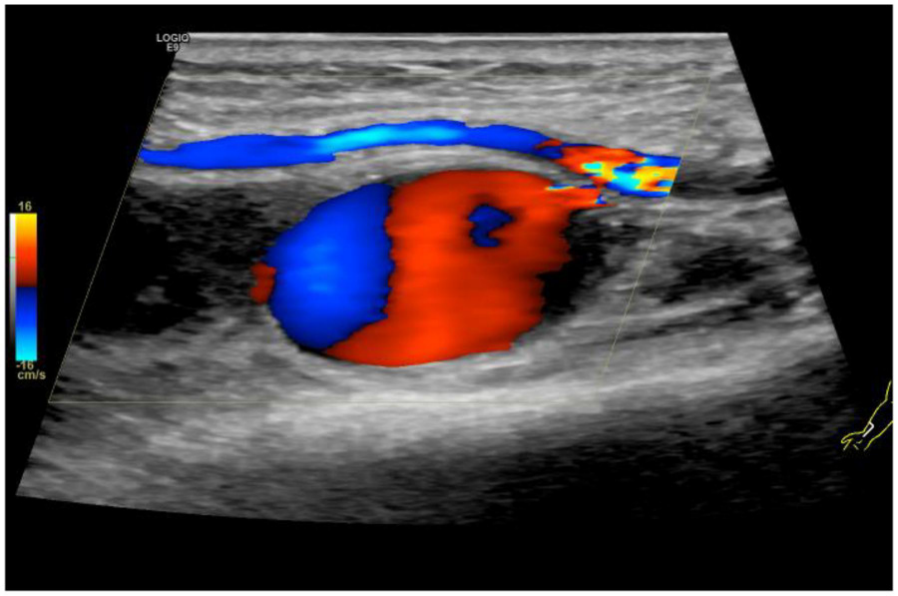
Doppler ultrasound of the right wrist was carried out, which confirmed the diagnosis of pseudoaneurysm of radial artery.

**Figure 2:**
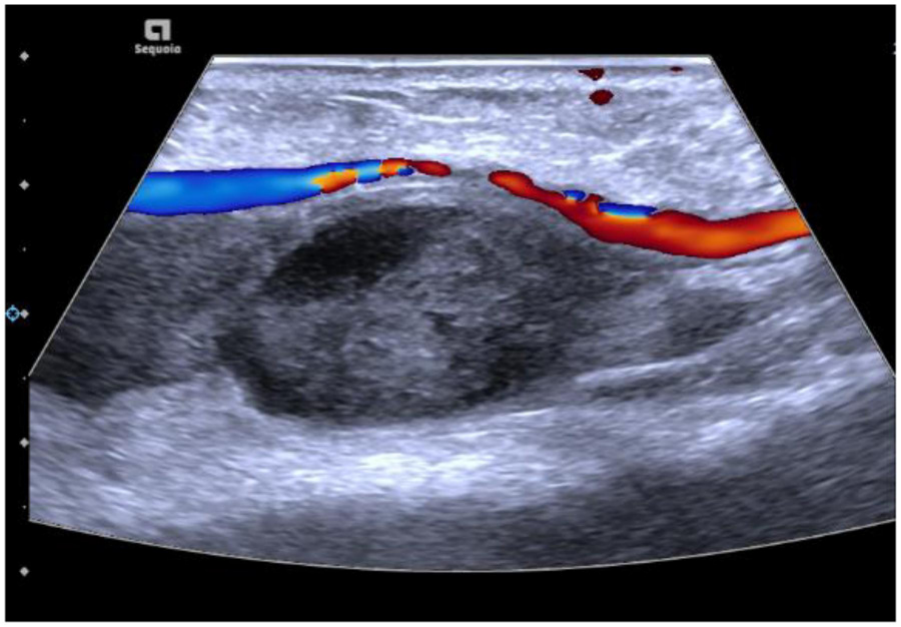
Ultrasound-guided injection of 1.5 mL of thrombin via a 21G needle. Immediate post-injection complete thrombosis on ultrasound imaging.

**Figure 3:**
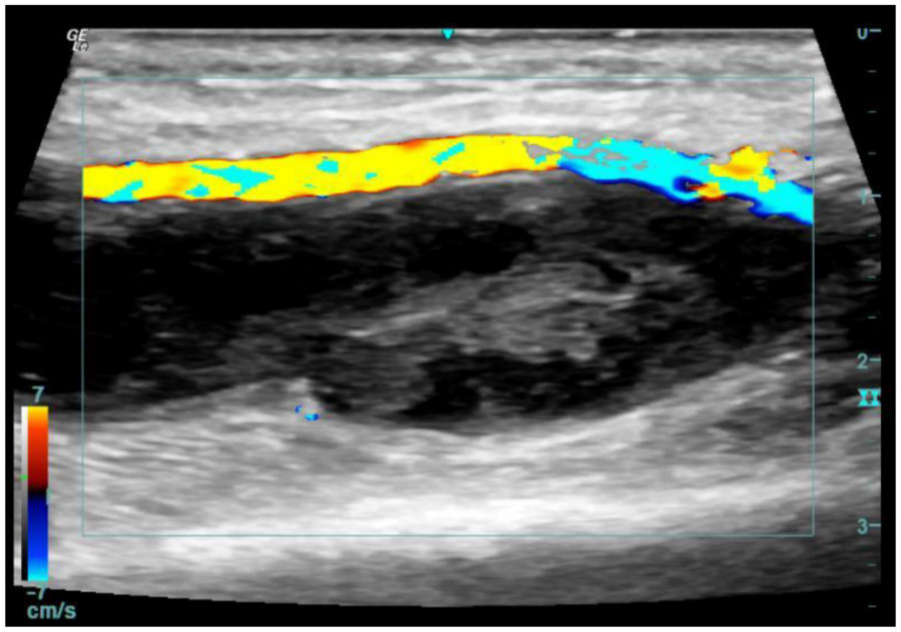
Ultrasound demonstrated sustained occlusion without evidence of recurrence at 24 h post-injection.

A 65-year-old Chinese male with a medical history of hypertension, dyslipidemia, and coronary artery disease was admitted due to unstable angina. Coronary angiography performed via right transradial access revealed significant triple-vessel disease, and PCI with drug-eluting stent implantation was successfully conducted. The procedure was uncomplicated, and hemostasis was achieved using a TR compression band device. On postoperative day 2, the patient reported swelling and tenderness at the right wrist. Physical examination identified a 2.5 × 2 cm pulsatile, tender mass with an audible bruit. Doppler ultrasonography confirmed the diagnosis of radial artery pseudoaneurysm, characterized by a narrow neck (2 mm) and a classic yin-yang flow pattern (Figure 1). After obtaining informed consent, UGTI was performed under continuous ultrasound guidance. A 21-gauge needle was used to inject 1.5 mL of purified human thrombin (500 IU/mL) into the pseudoaneurysm sac. Complete thrombosis occurred within 30 s (Figure 2). Post-procedurally, the mass became non-pulsatile, and follow-up ultrasound at 24 h confirmed sustained occlusion without recurrence (Figure 3). The patient remained asymptomatic and free of neurological or vascular complications.

## Discussion and conclusions

This case highlights the successful use of UGTI in managing iatrogenic RAP following transradial PCI, yet it also underscores the importance of appropriate patient selection and procedural nuance. Although RAP is uncommon, risk factors include advanced age, aggressive anticoagulation, and multiple puncture attempts. Traditional management options range from conservative measures such as prolonged external compression, often first-line for small, asymptomatic lesions, to surgical repair for larger or symptomatic aneurysms (12, 13).

As a superior alternative, UGTI offers a highly effective, minimally invasive approach, particularly in cases of narrow-necked pseudoaneurysms where compression therapy has failed. Critical technical considerations include precise needle placement within the aneurysm sac under continuous ultrasound guidance, avoiding injection into the neck or native artery to prevent distal thromboembolism (14). In our case, the pseudoaneurysm measured 2.5 × 2 cm with a 2 mm neck, making it suitable for UGTI. The procedure was performed by an interventional cardiologist with expertise in vascular ultrasound, in collaboration with a vascular radiologist, reflecting the multidisciplinary approach recommended for such interventions.

Despite its efficacy, UGTI is not without risks. Reported complications include thrombosis, distal embolism, allergic reactions to thrombin, and recurrence (11, 15). Therefore, UGTI should be considered after failure of conservative measures or when compression is deemed unlikely to succeed, rather than as a universal first-line option. Long-term follow-up is advised to monitor for recurrence, although most successfully thrombosed pseudoaneurysms remain stable.

In conclusion, UGTI represents a valuable minimally invasive strategy for managing eligible radial artery pseudoaneurysms, particularly those with narrow necks and symptomatic presentation. Its integration into clinical practice should be guided by lesion morphology, operator experience, and a balanced understanding of its risks and benefits, consistent with current guidelines that advocate for personalized, stepwise management of vascular complications.

## Data Availability

The original contributions presented in the study are included in the article/Supplementary Material, further inquiries can be directed to the corresponding author.
